# Subphenotyping depression using machine learning and electronic health records

**DOI:** 10.1002/lrh2.10241

**Published:** 2020-08-03

**Authors:** Zhenxing Xu, Fei Wang, Prakash Adekkanattu, Budhaditya Bose, Veer Vekaria, Pascal Brandt, Guoqian Jiang, Richard C. Kiefer, Yuan Luo, Jennifer A. Pacheco, Luke V. Rasmussen, Jie Xu, George Alexopoulos, Jyotishman Pathak

**Affiliations:** ^1^ Weill Cornell Medicine New York New York USA; ^2^ University of Washington Seattle Washington USA; ^3^ Mayo Clinic Rochester Minnesota USA; ^4^ Northwestern University Chicago Illinois USA

**Keywords:** depression, electronic health records, machine learning, phenotyping

## Abstract

**Objective:**

To identify depression subphenotypes from Electronic Health Records (EHRs) using machine learning methods, and analyze their characteristics with respect to patient demographics, comorbidities, and medications.

**Materials and Methods:**

Using EHRs from the INSIGHT Clinical Research Network (CRN) database, multiple machine learning (ML) algorithms were applied to analyze 11 275 patients with depression to discern depression subphenotypes with distinct characteristics.

**Results:**

Using the computational approaches, we derived three depression subphenotypes: Phenotype_A (n = 2791; 31.35%) included patients who were the oldest (mean (SD) age, 72.55 (14.93) years), had the most comorbidities, and took the most medications. The most common comorbidities in this cluster of patients were hyperlipidemia, hypertension, and diabetes. Phenotype_B (mean (SD) age, 68.44 (19.09) years) was the largest cluster (n = 4687; 52.65%), and included patients suffering from moderate loss of body function. Asthma, fibromyalgia, and Chronic Pain and Fatigue (CPF) were common comorbidities in this subphenotype. Phenotype_C (n = 1452; 16.31%) included patients who were younger (mean (SD) age, 63.47 (18.81) years), had the fewest comorbidities, and took fewer medications. Anxiety and tobacco use were common comorbidities in this subphenotype.

**Conclusion:**

Computationally deriving depression subtypes can provide meaningful insights and improve understanding of depression as a heterogeneous disorder. Further investigation is needed to assess the utility of these derived phenotypes to inform clinical trial design and interpretation in routine patient care.

## 1 INTRODUCTION

Clinical depression (depressive disorder) is one of the most common psychiatric disorders, which affects about 14% of individuals all over the world.^1^ The economic cost resulting from depression is staggering, which is expected to be the second largest contributor to disease burden by 2020.^2^ Clinical depression is a complex condition and patients with depression usually present a complex etiology, involving multiple risk factors such as recent stressful events.^3^, ^4^ In addition, clinical depression is usually associated with the elevated risk of other diseases such as cardiac diseases and mortality, including suicide.^5^ Furthermore, depression is highly recurrent in general populations.^6^ Therefore, the discovery of depression subphenotypes has a potential to improve the understanding of the underlying disease heterogeneity, which could provide benefits for patients in terms of early recognition and more targeted interventions and therapies. However, due to the complex etiology of depression, it is challenging to define depression subphenotypes based on clinical knowledge and empirical evidence.

Recently, the wider availability of Electronic Health Records (EHRs) has created a continuously growing repository of clinical data, which provides new opportunities for population‐based studies on a large scale and at low‐cost.^7^ Multiple data‐driven approaches for identifying disease phenotypes with EHRs have been explored.^8^, ^9^ From a data‐driven perspective, discovering phenotypes using EHRs can be seen as a “data clustering” problem.^9^, ^10^, ^11^ The disease manifestations of patients in the same cluster (ie, subphenotype) usually tend to be more similar. Comprehensive and longitudinal data captured in EHRs such as patient demographics, diagnoses, medications, laboratory measurements and procedures provide an opportunity to construct an appropriate representation for patients. The integration of these rich data and existing clustering methods such as hierarchical agglomerative clustering provide a potential to obtain clusters of patients, wherein each cluster corresponds to a unique subphenotype. Multiple statistical testing methods such as Chi‐square test^12^ can be performed on each cluster, which aim at finding discriminative variables across different clusters and providing interpretation for the computationally derived subphenotypes. The overall objective of this study is to define subphenotypes of depression disorders and investigate its clinical heterogeneity using machine learning methods and EHRs derived prior to patients' first case of depression. The ultimate goal is to provide assistance for the clinicians and further improve the ability to anticipate disease onset, for example, alert clinicians of the need for diagnostic work up for frequently co‐occurring disorders in those who fit the phenotype profile (Internists treating people for vascular risks and related disorders may suspect depression. Psychiatrists treating patients for depression may suspect vascular diseases or risk factors).

## 2 METHODS

### 2.1 Study data preparation

The INSIGHT Clinical Research Network (CRN) database^13^ was used to identify patients with depression between January 2008 and November 2017. This database includes records from more than 1 million patients consisting of demographics, comorbidities, encounters, procedures, medications, vital signs, and laboratory results. The INSIGHT CRN is the largest urban clinical network in the United States and reflects the racial, ethnic, and socioeconomic diversity of the national population as well as the range of healthcare facilities and services available in the United States. The collaborative EHR dataset compiles EHRs of 12 million patients from five large medical centers across New York City: Albert Einstein School of Medicine/Montefiore Medical Center, Columbia University and Weill Cornell Medicine/New York‐Presbyterian Hospital, Icahn School of Medicine/Mount Sinai Health System, Clinical Director's Network, and New York University School of Medicine/Langone Medical Center. Regarding the inclusivity and stability of the patient population, the INSIGHT CRN captures 471 federally qualified health centers, safety net clinics, primary care practices, and hospice centers staffed by 37 000 providers across the New York City metropolitan area. It also spans 11 years of longitudinal data on patients.

**FIGURE 1:**
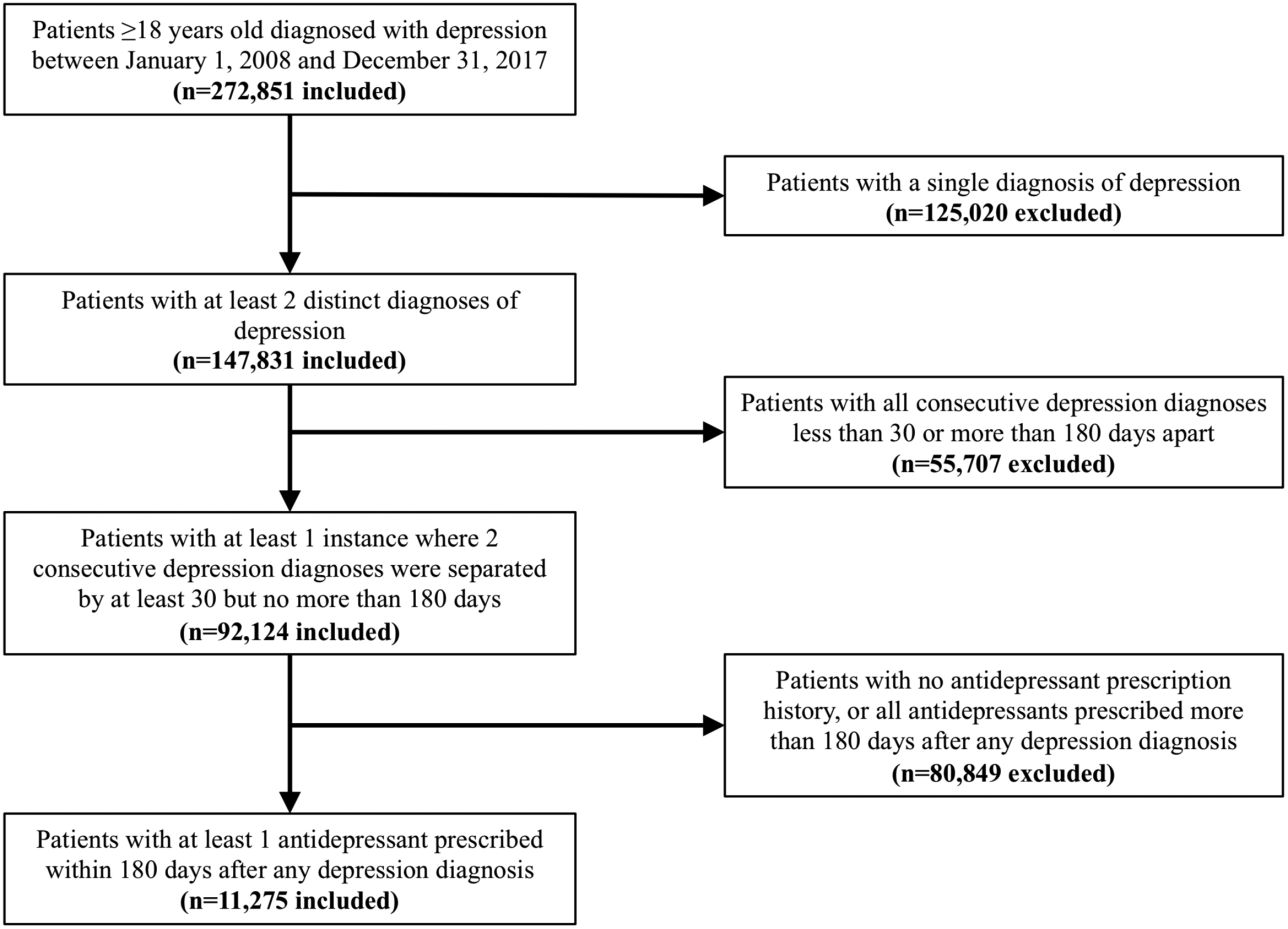
Exclusion cascade to identify the depression cohort from the INSIGHT CRN dataset

Figure 1 shows our exclusion cascade that led to the derivation of our “case” population of 11 275 patients who were diagnosed with depression and treated via pharmacotherapy. Patients aged 18 years and older were included if they had a gap of 30 to 180 days between 2 consecutive depression diagnoses and received an antidepressant 0 to 180 days after any depression diagnosis. We used 70 ICD9/10 codes (45.7% ICD9 codes, 54.3% ICD10 codes) for depression and a large number of RxNorm codes specific to antidepressant medication (Appendix S1).

In this study, we also identified a “control” population (1:1 ratio) matched on age, gender, and comorbidity using propensity risk scoring.^14^ To select the best control subject (non‐depressed patient) for each case subject (depressed patient), we used Nearest Neighbor Matching and matched covariates using the propensity score distance measure.^15^ The “control” group is used for model training and then obtains the best classifier that is used to choose important variables to perform clustering. The basic summary statistics of our dataset are shown in Table 1.

**TABLE 1:** Characteristics of case (depressed) and control (non‐depressed) groups

Item	Depressed (n = 11 275)	Non‐depressed (n = 11 275)
*Age* [mean (SD)]	62.6 (19.5)	63.7 (20.1)
18 to 24	234 (2.1%)	249 (2.2%)
25 to 44	2134 (18.9%)	2101 (18.6%)
45 to 64	3729 (33.1%)	3340 (29.6%)
≥65	5178 (45.9%)	5585 (49.5%)
*Gender*		
Female	7777 (69.0%)	7698 (68.3%)
*Race*		
White	3590 (31.8%)	2475 (22.0%)
Black or African American	981 (8.7%)	3260 (28.9%)
Asian	456 (4.0%)	253 (2.2%)
American Indian or Alaska Native	26 (0.2%)	39 (0.3%)
Native Hawaiian or Other Pacific Islander	17 (0.2%)	9 (0.1%)
*Ethnicity*		
Not Hispanic or Latino	6359 (56.4%)	8220 (72.9%)
Hispanic or Latino	1502 (13.3%)	631 (5.6%)

For this cohort, all demographic information (age, gender, race, and ethnicity) was extracted. Multiple comorbidities were also extracted based on the CMS Chronic Conditions Warehouse (CCW). Medication data was mapped to the Anatomical Therapeutic Chemical (ATC) Classification System,^16^ which classifies the active ingredients of drugs by taking into account their therapeutic, pharmacological and chemical properties. In the ATC system, drugs are classified into groups at five different levels. In this study, the fourth level was used to map medication information, which is usually more appropriate to identify pharmacological subgroups.^17^ All demographic, comorbidity and medication information were used to train the classifiers on multiple machine learning models. There are more than 500 features used for training machine learning models. We encoded medications and comorbidities as ever/never (1/0).

### 2.2 Classification and clustering

In order to choose multiple variables that are useful for discovering the subphenotypes, the “current classification” experimental setting^18^ was applied in this study. In particular, let *t* be the time of “first diagnosis” for depression either during an outpatient or inpatient encounter. In this setting, we considered all the data prior to time *t* and extracted patient demographics, comorbidities, and medications for training multiple machine learning models to classify depression. For each patient in the control group, the “time t” is the time of the last record of the patient in our dataset, which means we extracted all data for patients in the control group. Machine learning models included L2 norm regularized Logistic Regression (Ridge) ^19^, Random Forest (RF) ^20^, Support Vector Machine (SVM),^21^ and Gradient Boosting Decision Tree (GBDT). For each classification model, fivefold cross validation was adopted based on empirical knowledge. For Ridge, RF, SVM, we used the Scikit‐learn software library.^22^ For the GBDT, we chose XGBoost software library.^23^ The area under the receiver operating characteristic (AUC) was used to evaluate the model performance. Features from the model that performed the best, were ranked and ordered based on their variable importance measure, and subsequently used as inputs for the hierarchical agglomerative clustering algorithm to identify subphenotypes. We used the hierarchical agglomerative clustering algorithm from the Scikit‐learn software library.^22^ The only continuous variable (age) was excluded during this process, and similarity between the clusters was computed using the Jaccard Index. Clustergram^24^ was used to visualize the derived subphenotypes. Note that, during computing the similarity of patients using Jaccard Index method, we chose the patients who had at least 3 encounters in their historical records to minimize data sparsity. We finally chose 8930 patients for clustering. Multiple statistical analyses, such as Chi‐square test for binary variables and Kruskal‐Wallis H‐test for continuous variables with non‐normal distribution, were performed on experimental results to investigate the significance of features among clusters.

## 3 RESULTS

### 3.1 The performance of classification and obtaining the depression subphenotypes

**FIGURE 2:**
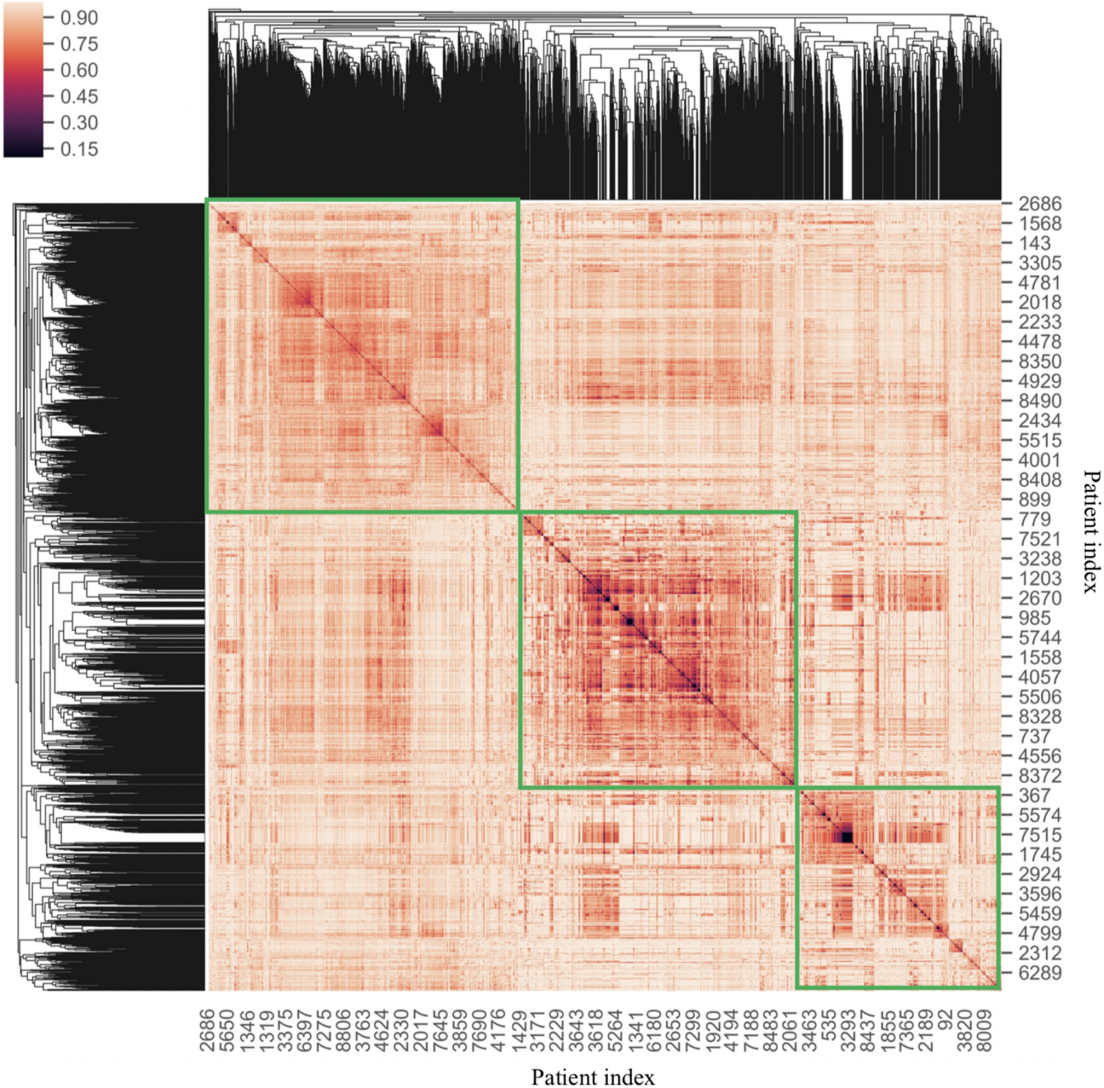
The heatmap obtained from Clustergram based on the selected variables. The *x* and *y* axis represents the patients' unique ID. The similarity among the individual patients was computed using the Jaccard Index. The “green rectangles” represent the three depression subphenotypes. The smaller the distance of patients were, the darker the color was, the greater the degree of similarity among patients were. The clusters can be approximately outlined on the clustermap by observing the distribution of colors along the diagonal line of the distance matrix

As shown in Table 2, GDBT achieved the highest performance for the current classification task in terms of AUC. By extracting feature importance scores from the GBDT model, we obtained multiple variables, including demographics, comorbidities and medications, with feature importance scores greater than zero. These variables were subsequently used as inputs for the clustering algorithm. By using Jaccard Index and hierarchical clustering, we obtained three depression subphenotypes (Figure 2). The optimal number of clusters was obtained by using the McClain index.^25^

**TABLE 2:** Performance of machine learning models for current classification of depression

	Precision	Recall	AUC
*L2 norm regularized Logistic Regression (Ridge)*	0.8511 ± 0.0078	0.6802 ± 0.0068	0.857 ± 0.0053
*Support Vector Machine (SVM)*	0.8855 ± 0.0088	0.5815 ± 0.0075	0.8376 ± 0.0052
*Random Forest (RF)*	0.6055 ± 0.0067	0.9074 ± 0.0072	0.8066 ± 0.0081
*Gradient Boosting Decision Tree (GBDT)*	0.8583 ± 0.0084	0.6919 ± 0.0097	**0.8711 ± 0.0058**

### 3.2 Association of comorbidities with the depression subphenotypes

**FIGURE 3:**
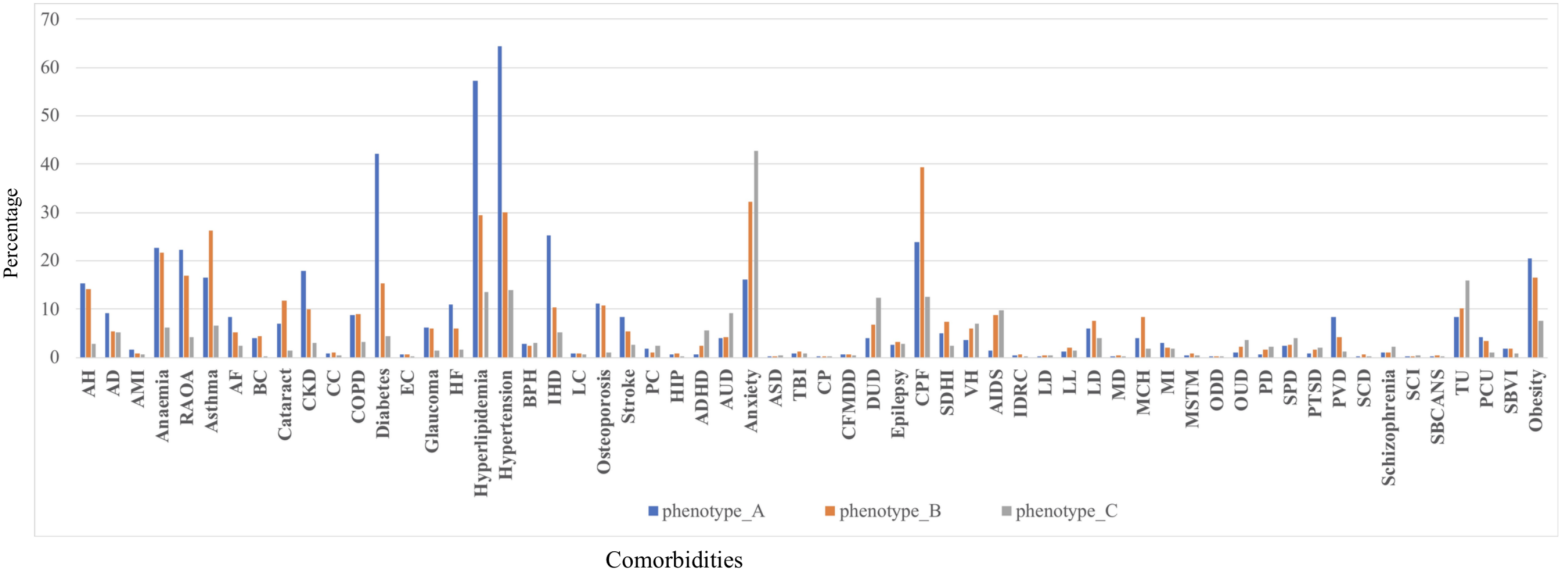
The percentage of patients with comorbidity in phenotypes. The *x* and *y* axis represent comorbidity and percentage, respectively. AH: Acquired Hypothyroidism; AD: Alzheimer's Disease and Related Disorders or Senile Dementia; AMI: Acute Myocardial Infarction; RAOA: Rheumatoid Arthritis/Osteoarthritis; AF: Atrial Fibrillation; BC: Breast Cancer; CKD: Chronic Kidney Disease; CC: Colorectal Cancer; COPD: Chronic Obstructive Pulmonary Disease and Bronchiectasis; EC: Endometrial Cancer; HF: Heart Failure; HIP: Hip/Pelvic Fracture; ADHD: Attention‐Deficit/Hyperactivity Disorder; AUD: Alcohol Use Disorders; ASD: Autism Spectrum Disorders; TBI: Traumatic Brain Injury and Nonpsychotic Mental Disorders due to Brain Damage; CP: Cerebral Palsy; CFMDD: Cystic Fibrosis and Other Metabolic Developmental Disorders; DUD: Drug Use Disorders; CPF: Chronic Pain and Fatigue, Fibromyalgia; SDHI: Sensory ‐ Deafness and Hearing Impairment; VH: Viral Hepatitis; AIDS: Acquired Immunodeficiency Syndrome; IDRC: Intellectual Disabilities and Related Conditions; LD: Learning Disabilities; LL: Leukemias and Lymphomas; LD: Liver Disease; MD: Muscular Dystrophy; MCH: Migraine and Chronic Headache; MI: Mobility Impairments; MSTM: Multiple Sclerosis and Transverse Myelitis; ODD: Other Developmental Delays; OUD: Opioid Use Disorder; PD: Personality Disorders; SPD: Schizophrenia and Other Psychotic Disorders; PTSD: Post‐Traumatic Stress Disorder; PVD: Peripheral Vascular Disease; SCD: Sickle Cell Disease; SCI: Spinal Cord Injury; SBCANS: Spina Bifida and Other Congenital Anomalies of the Nervous System; TU: Tobacco Use; PCU: Pressure and Chronic Ulcers; SBVI: Sensory—Blindness and Visual Impairment

Figure 3 shows the distribution of comorbidities across all three subphenotypes. We observe that patients in Phenotype_A and Phenotype_C had the highest and lowest number of comorbidities, respectively. In particular, within Phenotype_A, cardiovascular conditions such as hyperlipidemia (57.18%), hypertension (64.41%), and diabetes (42.17%) were commonly observed. In Phenotype_B, most patients suffered from asthma (26.22%) and chronic pain and fatigue (39.29%), whereas in Phenotype_C, anxiety (42.7%) and tobacco use (15.96%) was commonly observed. From this table, we also observe that Phenotype_B had the most number of patients (n = 4687) accounting for more than half of all patients. The average age in this subphenotype was 68.44 ± 19.09 years. Patients in Phenotype_A and Phenotype_C are the oldest and the youngest, respectively. Note that, there is no significance (*P*‐value >.05) in terms of age among these three phenotypes. In addition, in Phenotype_A and Phenotype_B, the number of females is nearly twice the number of males.

### 3.3 Association of medications with the depression subphenotypes

**FIGURE 4:**
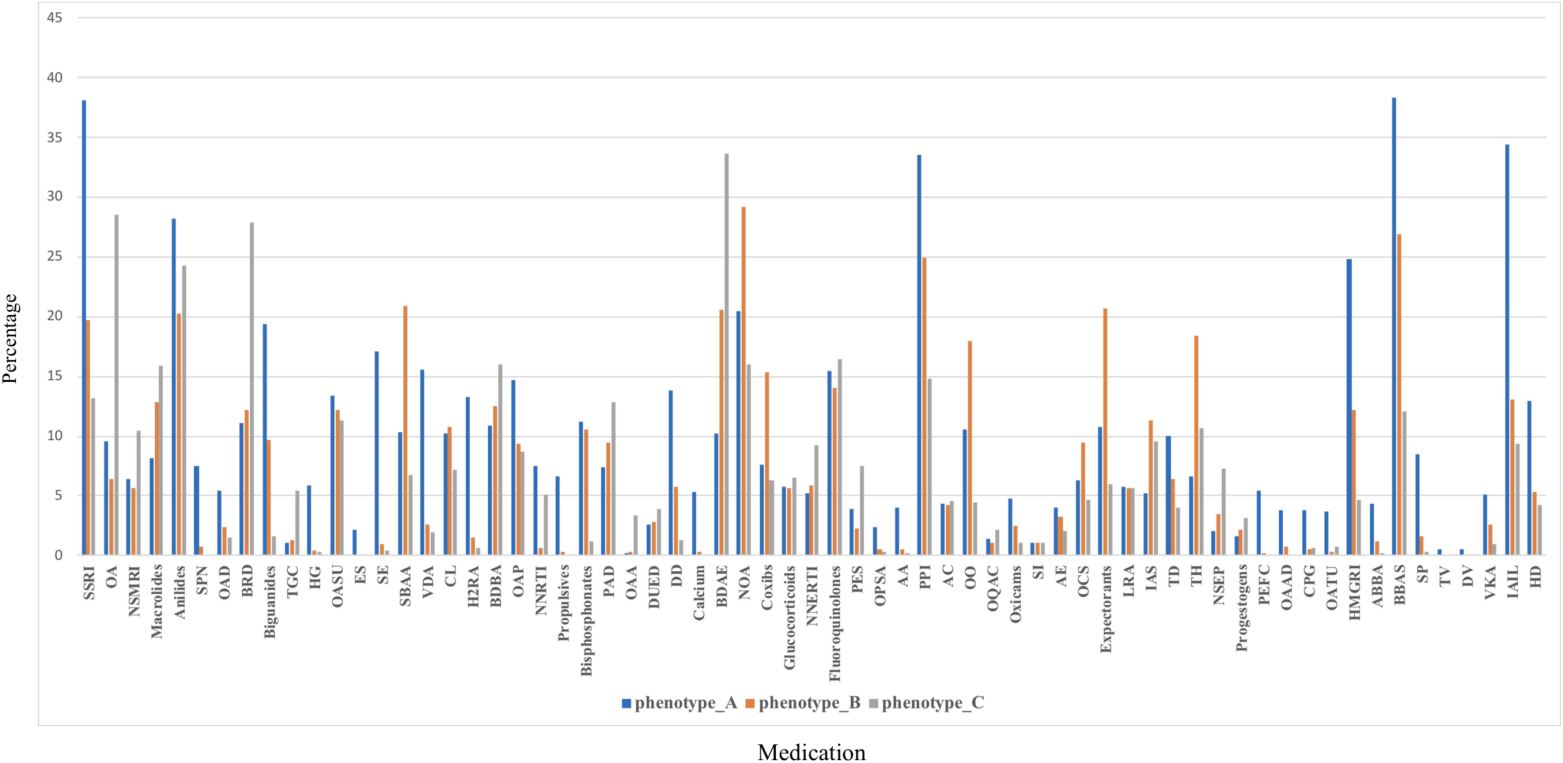
The percentage of patients with medications in phenotypes. The *x* and *y* axis represent medication and percentage, respectively. SSRI: Selective Serotonin Reuptake Inhibitors; OA: Other Antidepressants; NSMRI: Non‐Selective Monoamine Reuptake Inhibitors; SPN: Solutions for Parenteral Nutrition; OAD: Opium Alkaloids and Derivatives; BRD: Benzodiazepine Related Drugs; TGC: Third‐Generation Cephalosporins; HG: Heparin Group; OASU: Other Antihistamines for Systemic Use; ES: Electrolyte Solutions; SE: Softeners Emollients; SBAA: Selective Beta‐2‐Adrenoreceptor Agonists; VDA: Vitamin D and Analogues; CL: Contact Laxatives; H2RA: H2‐Receptor Antagonists; BDBA: Benzodiazepine Derivatives (N05BA); OAP: Other Antiepileptics; NNRTI: Nucleoside and Nucleotide Reverse Transcriptase Inhibitors; PAD: Propionic Acid Derivatives; OAA: Oxytocin and Analogues; DUED: Drugs Used in Erectile Dysfunction; DD: Dihydropyridine Derivatives; BDAE: Benzodiazepine Derivatives (N03AE); NOA: Natural Opium Alkaloids; NNERTI: Nucleosides and Nucleotides Exclude Reverse Transcriptase Inhibitors; PES: Penicillins with Extended Spectrum; OPSA: Other Potassium‐Sparing Agents; AA: Aldosterone Antagonists; PPI: Proton Pump Inhibitors; AC: Aluminium Compounds; OO: Other Opioids; OQAC: Other Quaternary Ammonium Compounds; SI: Selective Immunosuppressants; AE: Aminoalkyl Ethers; OCS: Other Cough Suppressants; LRA: Leukotriene Receptor Antagonists; IAS: Intermediate‐Acting Sulfonamides; TD: Trimethoprim and Derivatives; TH: Thyroid Hormones; NSEP: Natural and Semisynthetic Estrogens Plain; PEFC: Progestogens and Estrogens, Fixed Combinations; OAAD: Other Antiseptics and Disinfectants; CPG: Corticosteroids, very Potent (group IV); OATU: Other Antibiotics for Topical Use; HMGRI: HMG CoA Reductase Inhibitors; ABBA: Alpha and Beta Blocking Agents; BBAS: Beta Blocking Agents, Selective; SP: Sulfonamides Plain; TV: Tetanus Vaccines; DV: Diphtheria Vaccines; VKA: Vitamin K Antagonists; IAIL: Insulins and Analogues for Injection, Long‐acting; HD: Hydrazinophthalazine Derivatives

Figure 4 shows the distribution of medications across all three subphenotypes. In general, we observe that patients in Phenotype_A and Phenotype_C took more and fewer medications, respectively. In particular, patients in Phenotype_A had higher rates of prescriptions for antidepressants, beta blockers, and insulin. In Phenotype_B, we observed higher rates of prescriptions for opioids, proton pump inhibitors, and adrenergic β2 receptor agonists. Finally, for Phenotype_C, benzodiazepines were the most commonly prescribed medication.

In addition, to further investigate the characteristics of three subphenotypes, we performed multiple statistical analyses on our results. As shown in Table 3, the results indicate that comorbidities including hypertension, hyperlipidemia, diabetes, asthma, chronic pain and fatigue, and tobacco use were significantly different across the subphenotypes. Similarly, the results suggest that use of antidepressants, insulin, opioids, adrenergic β2 receptor agonists and benzodiazepines was significantly different across the subphenotypes.

**TABLE 3:** Characteristics of the three depression subphenotypes

Characteristic	Phenotypes			Unadjusted *P*‐value	Adjusted ANCOVA^a^
	A	B	C		
No. of patients (%) Total (8903 patients)	2791 (31.35)	4687 (52.65)	1452 (16.31)		
*Age*, Mean (SD)	72.55 (14.93)	68.44(19.09)	63.47(18.81)	.526	‐
*Sex*, No. (%)					
Female	1716 (61.48)	3341 (71.29)	866 (59.66)	.456	0.643
Male	1075 (38.52)	1346 (28.71)	586 (40.34)		
*Comorbidity*, No. (%)					
Hypertension	1798 (64.41)	1403 (29.93)	203 (13.96)	≤.05	≤0.05
Diabetes	1176 (42.17)	714 (15.23)	64 (4.42)	≤.05	≤0.05
Hyperlipidemia	1596 (57.18)	1379 (29.42)	198 (13.61)	≤.05	≤0.05
RAOA	623 (22.32)	788 (16.81)	60 (4.14)	.213	0.368
Anemia	632 (22.64)	1014 (21.63)	90 (6.18)	.564	0.482
Asthma	459 (16.45)	1229 (26.22)	96 (6.6)	≤.05	≤0.05
CPF	667 (23.9)	1842 (39.29)	181 (12.49)	≤.05	≤0.05
Anxiety	448 (16.05)	1564 (32.13)	620 (42.7)	≤.05	≤0.05
TU	231 (8.28)	478 (10.2)	232 (15.96)	≤.05	≤0.05
Obesity	572 (20.49)	769 (16.41)	111 (7.65)	.642	0.775
*Drugs*, No. (%)					
Selective serotonin reuptake inhibitors	1064 (38.11)	926 (19.75)	191 (13.12)	≤.05	≤0.05
Beta blocking agents, selective	1069 (38.3)	1262 (26.92)	176 (12.11)	.321	0.535
Insulins and analogues for injection, long‐acting	961 (34.42)	613 (13.08)	136 (9.4)	≤.05	≤0.05
Natural opium alkaloids	571 (20.46)	1370 (29.22)	232 (15.98)	≤.05	≤0.05
Proton pump inhibitors	935 (33.5)	1171 (24.98)	214 (14.77)	.225	0.327
Selective beta‐2‐adrenoreceptor agonists	290 (10.38)	980 (20.9)	98 (6.77)	≤.05	≤0.05
Benzodiazepine derivatives	287 (10.28)	963 (20.54)	489 (33.65)	≤.05	≤0.05
Benzodiazepine related drugs	310 (11.12)	571 (12.19)	406 (27.93)	.381	0.499
Other antidepressants	266 (9.54)	300 (6.41)	415 (28.59)	.568	0.768
Expectorants	302 (10.82)	967 (20.64)	87 (5.98)	≤.05	≤0.05

Abbreviations: CPF, Chronic Pain and Fatigue, Fibromyalgia; RAOA, Rheumatoid Arthritis/Osteoarthritis; TU, Tobacco Use.

^a^ ANCOVA was performed to adjust significance in terms of age variable. The only continuous variable age is tested by using Kruskal‐Wallis H‐test. Other binary variables are tested by using Chi‐square test.

## 4 DISCUSSION

Three distinct depression subphenotypes were computationally derived from EHR data including patient demographics, comorbidities and medications using machine learning methods. Among the derived subphenotypes, statistically significant differences were observed with respect to disease burden and medication prescriptions. Such an approach provides an opportunity to improve our understanding of a heterogeneous disorder such as depression, and potentially enables improved diagnosis and treatment.

In particular, across the three depression subphenotypes, patients in Phenotype_A (n = 2791; 31.35%) were relatively older (mean (SD) age, 72.55 (14.93) years), had the highest number of vascular comorbidities and took the most number of medications. These results are consistent with previous reports. For example, prior studies have shown that depression was two to three times more likely in people with multimorbidity compared to people without multimorbidity or those who have no chronic physical condition.^26^ Hypertension may be an important factor for patients with depression in this group. For example, a population‐based study in Stockholm County, Sweden demonstrated that hypertension was probably underdiagnosed and ignored in individuals with psychiatric disorders.^27^ Multiple studies have also suggested that the risk of developing depression was increased in individuals with diabetes^28^ and that there was significant association between depression and diabetes.^29^ The connections between depression and hyperlipidemia have also been shown^30^ and few studies have suggested that preexisting hyperlipidemia could be an independent predictor of new‐onset depression.^31^ In our study, Phenotype_C (n = 1452; 16.31%) was the youngest (mean (SD) age, 63.47 (18.81) years) and included the least number of patients with fewer comorbidities and prescription medications. Furthermore, the comorbidities of anxiety and tobacco use were common in this subphenotype. Patients in this subphenotype also showed mild loss of their body function. Strong associations exist between depression and anxiety and previous studies have suggested that more than 50% of patients with an anxiety disorder had depression.^32^ An association between tobacco use and depression has also been shown by multiple previous studies^33^, ^34^, ^35^ and cigarette use was positively associated with depressive symptoms among young people such as college students.^36^ Phenotype_B (n = 4687; 52.65%) included most patients (mean (SD) age, 68.44 (19.09) years) in our study. The common comorbidities in this subphenotype were asthma and chronic pain and fatigue. Associations between asthma and clinically significant levels of depressive symptoms and a lifetime psychiatric disorder have been reported in several studies.^37^, ^38^ The relationship between depression and chronic pain have been shown in previous studies,^39^ which considered multiple factors such as inflammatory, infectious, and autoimmune disorders in terms of the development of fibromyalgia. Identifying depressive subtypes with distinct patterns of medical comorbidity may help to generate hypotheses on the etiopathogenesis of late‐life depressive syndromes and provide targets for treatment development.^40^

Few studies have also investigated the identification of depression subphenotypes using multimodal data.^41^, ^42^ For example, Drysdale et al^10^ used functional magnetic resonance imaging (fMRI) data and machine learning algorithms to detect four neurophysiological depression subtypes defined by distinct patterns of dysfunctional connectivity in limbic and frontostriatal networks. Tokuda et al^43^ also used fMRI data to detect three neurophysiological subtypes of depression that related to Selective Serotonin‐Reuptake Inhibitor (SSRI) treatment outcomes. Musil et al^44^ used the DSM‐IV specifiers on a cohort of 833 patients to manually classify melancholic, atypical and anxious subtypes of depression. There are few key differences between these studies and our study. First, our work leverages routinely collected EHRs from multiple health systems in an urban population. Such data, while not pristine compared to curated datasets from clinical trials or prospective studies, reflects actual clinical care, including diagnosis and treatment. Second, at least to our knowledge, our study cohort of more than 20 000 subjects (cases and controls) to detect depression subphenotypes is one of the largest to date. And finally, we demonstrate the applicability of off‐the‐shelf machine learning algorithms for subphenotyping which provides a more interpretable and generalizable framework for implementing our approach in external datasets for future replication studies.

However, the results of our study should be considered in light of several limitations. First, it should be noted that this study examines a niche group of depressed patients who were treated via pharmacotherapy within a very narrow time window. During the 2008 to 2017 time span of observation, detection standards for depression are not well defined and documentation routines are highly variable.^45^, ^46^, ^47^ In addition, off‐label use of antidepressants is common in treating sleep problems, eating disorders, smoking cessation, and managing chronic pain even when depression is not involved.^48^ By restricting the study cohort to depressed patients treated via pharmacotherapy, we might be missing patients whose prescription data is not captured in the INSIGHT CRN. It is possible that many of these patients received an antidepressant from a private provider outside the INSIGHT CRN network or received alternative therapies such as psychotherapy or cognitive behavioral therapy (CBT) to treat their depressive symptoms. Unfortunately, our dataset is unable to capture these treatment modalities. It is also possible that patients initiated alternative treatments like psychotherapy and CBT during the 0 to 180 day time window but later transitioned into treatment via pharmacotherapy (eg, antidepressant). With careful consideration given to limitations including a dramatically smaller cohort, we selected a highly sensitive case definition that minimizes the inclusion of false positives and ensures a highly chronic dual diagnosis sample. Second, we only considered patient demographics, diagnoses, and prescription medication data extracted from the EHR for deriving the subphenotypes. Prior work by others^49^ and our team^50^ has demonstrated that for mood disorders, processing of unstructured clinical text via natural language processing is critical to detect symptoms, diagnosis and treatment. Third, we did not consider temporal information (eg, age of disease onset) for our classification and clustering tasks. Temporal data may correspond to a patient's current therapy, their overall health status, or any other discrete state, and the transition time information represents the duration of each of those states. In future work, we plan to leverage recent research in temporal pattern mining for clustering analysis.^51^, ^52^ Finally, with an emphasis on algorithm interpretation, portability and generalizability, we investigated traditional machine learning algorithms in this study. As we have done in other studies,^9^, ^53^, ^54^ future work will explore advanced deep learning methods for depression subphenotyping.

## 5 CONCLUSION

Using routinely collected longitudinal EHRs and ML algorithms, we computationally derived depression subphenotypes that can potentially guide improved diagnosis and treatment of clinical depression. The derived subphenotypes had statistically significant differences with respect to patient demographics, comorbidities and treatment suggesting that depression is a heterogeneous disorder with multiple phenotypes.

## Supporting information


**Appendix** 1 Supporting Information
