# Supplementary material for: Subphenotyping depression using machine learning and electronic health records
Source: Learn Health Syst. 2020 Aug 3;4(4):e10241. doi: 10.1002/lrh2.10241 (PMC7556423; doi:10.1002/lrh2.10241)
Supplement: Supplementary file 1 — Appendix 1 Supporting Information [file LRH2-4-e10241-s001.docx]

| **Table 4 The ICD9, ICD10 codes and Antidepressant RxNorm**  **codes** | | |
| --- | --- | --- |
| **Item** | | **Codes** |
| **Diagnosis Codes** | **ICD9 codes** | 296.20; 296.21; 296.22; 296.23; 296.24; 296.25; 296.26; 296.30; 296.31; 296.32; 296.33; 296.34; 296.35; 296.36; 296.51; 296.52; 296.53; 296.54; 296.55; 296.56; 296.60; 296.61; 296.62; 296.63; 296.64; 296.65; 296.66; 296.89; 298.0; 300.4; 309.1; 311 |
|  | **ICD10 codes** | F31.30; F31.31; F31.32; F31.4; F31.5; F31.60; F31.61; F31.62; F31.63; F31.64; F31.75; F31.76; F31.77; F31.78; F31.81; F32; F32.0; F32.1; F32.2; F32.3; F32.4; F32.5; F32.9;F33.0; F33.1; F33.2; F33.3; F33.40; F33.41; F33.42; F33.8; F33.9; F34.1; F34.8; F34.9; F39.0; F43.21; F43.23 |
| **Antidepressant RxNorm**  **Codes** | **Amitriptyline** | 704; 856706; 856720; 856783; 856762; 856769; 856773; 856792; 856797; 856825; 856834; 856840; 856845; 856853 |
|  | **Amoxapine** | 197363; 197364; 197365; 197366 |
|  | **Bupropion** | 42347; 993687; 993688; 993691; 993693; 993503; 993505; 993511; 993518; 993520; 993528; 993536; 993537; 1551468; 1551474; 993550; 993552; 993567; 993569; 993681; 993683; 993541; 993542; 993545; 993557; 993564; 1232585; 1232591; 1801289; 993524; 993954 |
|  | **Citalopram** | 2556; 283672; 284591; 309313; 200371; 213344; 309314; 213345 |
|  | **Clomipramine** | 857297; 857299; 857301; 857303; 857305 |
|  | **Desipramine** | 3247; 1099288; 1099292; 1099296; 1099300; 1099304; 1099316 |
|  | **Desvenlafaxine** | 790264; 790288; 790267; 1607617; 1607619; 790290 |
|  | **Doxepin** | 966787; 966791; 966793; 966795; 1000048; 1000054; 1000058; 1000064; 1000070; 1000076; 1000091; 1000093; 1000095; 1000097 |
|  | **Duloxetine** | 72625; 596926; 596928; 596930; 596932; 616402; 596934; 615186 |
|  | **Escitalopram** | 321988; 351285; 404420; 349332; 352272; 351250; 352273; 351249; 404408 |
|  | **Fluoxetine** | 4493; 310384; 205535; 313990; 647555; 310385; 104849; 248642; 647556; 403970; 721787; 725080; 403969; 725064; 310386; 313989; 261287; 403971; 403972; 725072; 1190110; 313995; 598032; 903873; 903875; 903879; 903881; 903884; 903887; 903891 |
|  | **Imipramine** | 5691; 835593; 835564; 835566; 835568; 835570; 835591; 835598; 835589; 835577; 835572; 835574 |
|  | **Isocarboxazid** | 104837 |
|  | **levomilnacipran** | 1433249; 1433217; 1433223; 1433227; 1433229; 1433233; 1433235; 1433239; 1433241 |
|  | **Maprotiline** | 6646; 1298857; 1298861; 1298870 |
|  | **Mirtazapine** | 15996; 283406; 752869; 311725; 211322; 283407; 752870; 314111; 211323; 283485; 311726; 261135; 476809 |
|  | **Nefazodone** | 1098649; 1098666; 1098670; 1098674; 1098678 |
|  | **Nortriptyline** | 7531; 198045; 209329; 312036; 317136; 209339; 198046; 209350; 198047 |
|  | **Paroxetine** | 1738803; 1738804; 1738805; 1738806; 1738807; 1738808; 898697; 32937; 1738483; 211699; 312242; 213291; 1738495; 207349; 1738503; 207350; 1738511; 211700; 1738515; 541660; 1738519; 541662; 1738523; 541664; 1738527; 541666; 1430122; 1430128 |
|  | **Phenelzine** | 312347; 104836 |
|  | **Protriptyline** | 905168; 905170; 905172; 905174 |
|  | **Selegiline** | 859190; 859192; 859186; 859193 |
|  | **Sertraline** | 36437; 312938; 208149; 861064; 861066; 312940; 212233; 312941; 208161 |
|  | **Tranylcypromine** | 10734; 313447; 104838 |
|  | **Trazodone** | 10737; 898701; 898704; 898706; 856373; 856364; 856369; 856377 |
|  | **Trimipramine** | 313496; 313498; 313499 |
|  | **Venlafaxine** | 313581; 729931; 808744; 808748; 313583; 729932; 808751; 313585; 729929; 808753; 39786; 313580; 313582; 313584; 314277; 208851; 313586 |
|  | **Vilazodone** | 1086790; 1653469; 1086789; 1086772; 1086776; 1086778; 1086780; 1086784; 1086786 |
|  | **Vortioxetine** | 1439808; 1439844; 1790886; 1439810; 1439838; 1790890; 1439812; 1439829; 1790892 |
